# SARS-CoV-2 in the abdomen or pelvis: SAFE SURGERY study

**DOI:** 10.1093/bjs/znac297

**Published:** 2022-09-28

**Authors:** Dale Vimalachandran, Robert P Jones, Ed Dickson, Jaspreet Seehra, Austin Acheson, Ewen A Griffiths, Sivesh Kamarajah, Elaine Leung, Andrew Torrance, Christian Ottensmeier, Andrew D Beggs, Ellen Whiteside, Helen Sanna, Danielle Bury, Esther Youd, Gareth Leopold, Matthew Pugh, Sudha Sundar, Graham S Taylor

**Affiliations:** Department of General and Colorectal Surgery, Countess of Chester NHS Trust, Chester, UK; Department of General and Hepatobiliary Surgery, Liverpool University Hospital NHS Trust, Liverpool, UK; Department of General and Colorectal Surgery, Nottingham University Hospitals NHS Trust, Nottingham, UK; Department of General and Colorectal Surgery, Nottingham University Hospitals NHS Trust, Nottingham, UK; Department of General and Colorectal Surgery, Nottingham University Hospitals NHS Trust, Nottingham, UK; Upper GI Unit, University Hospitals Birmingham NHS Trust, Birmingham, UK; Upper GI Unit, University Hospitals Birmingham NHS Trust, Birmingham, UK; Institute of Cancer and Genomic Sciences, University of Birmingham, Birmingham, UK; Department of General and Colorectal Surgery, Sandwell and West Birmingham NHS Trust, City Hospital,, Birmingham, UK; Liverpool Head and Neck Centre, Institute of Systems, Molecular and Integrative Biology, University of Liverpool, Liverpool, UK; Upper GI Unit, University Hospitals Birmingham NHS Trust, Birmingham, UK; Institute of Cancer and Genomic Sciences, University of Birmingham, Birmingham, UK; Department of Molecular Pathology, Blackpool Victoria Hospital NHS Trust, Blackpool, UK; Department of Molecular Pathology, Blackpool Victoria Hospital NHS Trust, Blackpool, UK; Department of Molecular Pathology, Blackpool Victoria Hospital NHS Trust, Blackpool, UK; Department of Pathology, Royal Glamorgan Hospital, Cwm Taf University Health Board, Llantrisant, UK; Department of Cellular Pathology, Morriston Hospital, Swansea Bay University Health Board, Swansea, UK; Institute of Immunology and Immunotherapy, University of Birmingham, Birmingham, UK; Institute of Cancer and Genomic Sciences, University of Birmingham, Birmingham, UK; Institute of Immunology and Immunotherapy, University of Birmingham, Birmingham, UK

## Introduction

As the global community recovers from the SARS-CoV-2 pandemic, the ability to return to normal clinical practice to relieve large clinical backlogs is critical. However, concerns over the risk of SARS-CoV-2 transmission to healthcare staff via surgery remain^1^.

The angiotensin-converting enzyme (ACE) 2 protein receptor, through which SARS-CoV-2 infects cells, is expressed by cells across multiple organs^2^. Small single-centre studies^3,4^ have reported the presence of the virus in the surgical plume or peritoneal fluid, whereas others^5–8^ have not. In an emergency surgery setting, concern has been raised where there has been a breach in the gastrointestinal tract, which may provide an additional route for the virus to reach the peritoneal cavity^9,10^.

To investigate this, a clinical study (SAFE SURGERY) was designed to assess the presence of SARS-CoV-2 in the abdominal cavity and organs of infected patients. SAFE SURGERY operated as three substudies. Substudy 1 was a multicentre, prospective study of peritoneal, rectal, and vaginal swabs from patients undergoing emergency abdominal or pelvic surgery who tested positive for SARS-CoV-2. Substudy 2 was a multicentre, retrospective analysis of archived surgical tissues from patients who tested positive for SARS-CoV-2, and who had previously undergone emergency abdominal or pelvic surgery. Substudy 3 comprised a multicentre analysis of post-mortem tissues from patients who had died from SARS-CoV-2 infection.

## Methods

Ethical approval for substudies 1 and 2 was obtained from the South Oxford B Research Ethics Committee (20/SC/0261). For substudy 3, ethical approval for sample collection was obtained from the Newcastle and North Tyneside Research Ethics Committee (REC: 19/NE/0336).

### Substudy 1

Patients were recruited from five centres across the UK (Chester, Liverpool, Nottingham, Birmingham, Sandwell) between 23 July 2020 and 30 March 2021. Patients were eligible if they were undergoing emergency surgery following a recent diagnosis of SARS-CoV-2 infection (PCR-positive or chest X-ray suggestive of infection). All patients had swabs taken from the pelvis and paracolic gutters either via a laparotomy wound or a laparoscopic port, along with repeat nasopharyngeal and luminal (rectal, colonic or vaginal) swabs. Samples were placed in viral transport medium and sent to the University of Birmingham within 24 h. Samples were inactivated by heating to 65°C for 30 min in a temperature-mapped oven, and RNA was extracted using a Qiagen RNeasy® (Manchester, UK) kit with a modified protocol. SARS-CoV-2 RNA was measured by reverse transcriptase–PCR using the CERTEST ViaSure SARS-CoV-2 real-time PCR kit against the *ORF1ab* and *N1* genes of the SARS-CoV-2 genome, as described previously^11^.

### Substudy 2

Local study teams identified patients who had undergone emergency surgery or had died after a positive respiratory PCR swab. Retrospective samples were identified and relevant tissue blocks were sent to Blackpool Victoria Hospital for pathological examination. Haematoxylin and eosin-stained slides were produced for each tissue block to look for the presence of mesothelium-lined fatty tissue (indicative of the peritonealized surface) within the block.

### Substudy 3

Patients were included if they had received a positive PCR swab, either in life or at post-mortem examination, and if COVID-19 was noted as the cause of death. Tissue samples were obtained from multiple organs and fixed for 72 h before processing and paraffin embedding. Wide-bore tissue cores were taken from each patient and used to construct tissue microarrays.

### Immunohistochemistry

For substudies 2 and 3, routine immunohistochemical (IHC) methods were used to stain for SARS-CoV-2 nucleocapsid protein (antibody clone ER81L, 1 : 200 dilution; Cell Signalling Technology) and ACE-2 receptor (antibody clone E-11, 1 : 100 dilution; Santa Cruz Biotechnology) using the Ventana Benchmark XT platform. Placental tissue from a patient known to be SARS-CoV-2-positive (*Fig. S1A*) and normal small bowel mucosa were used as positive controls for nucleocapsid and ACE-2 (*Fig. S1B*) respectively.

## Results

### Substudy 1: prospective swabs from surgical patients

Ten patients were recruited; 9 had positive nasopharyngeal swabs and 1 had clinical suspicion of infection based on a positive chest X-ray. Three of these patients underwent obstetric/gynaecological procedures and 7 had gastrointestinal surgery (*Table 1*). Repeat nasopharyngeal swabs were positive in 9 patients. Peritoneal swabbing was performed in all 10 patients; swabs were taken from the vagina in 1 patient, and from the colonic lumen in 1 patient. A total of 23 peritoneal, vaginal, and colonic swabs were taken, and all were negative for SARS-CoV-2 by PCR.

**Table 1:** Clinical and surgical characteristics of patients in substudy 1, and peritoneal swab results

	Substudy 1 (*n* = 10)	
**Age (years), median (range)**	46 (32–72)	
**Sex ratio (M : F)**	6 : 4	
**Positive PCR**	9 (1 positive CXR only)	
**Surgical approach**		
Open	2	
Laparoscopic	8	
**Prospective peritoneal swab**	***n***	**SARS-CoV-2 PCR**
Salpingectomy	1	Negative
Caesarean section	2	Negative
Colonic resection	4	Negative
Appendicectomy	1	Negative
Hepatectomy	1	Negative
Adhesiolysis	1	Negative

CXR, chest X-ray.

### Substudy 2: retrospective tissue samples from surgical patients

**Fig. 1:**
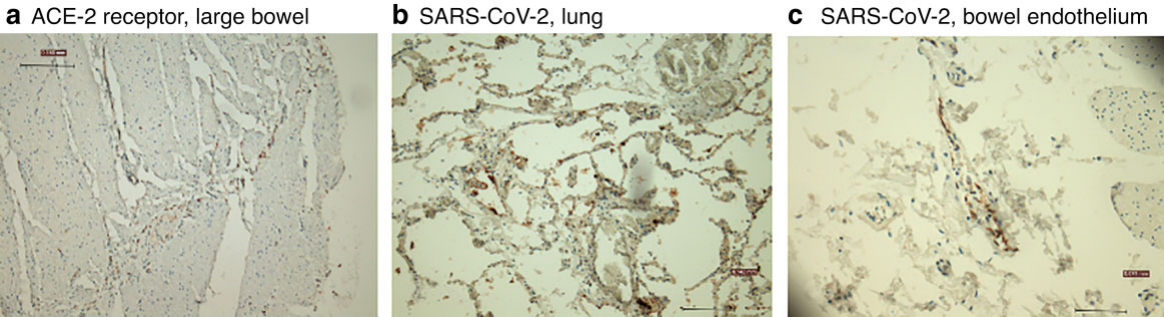
Examples of immunohistochemical staining **a** Immunohistochemical (IHC) staining showing faint angiotensin-converting enzyme (ACE) 2 receptor expression in large bowel from a patient in substudy 2 (ACE-2 antibody, Santa Cruz Biotechnology; ×100 magnification); **b** positive SARS-CoV-2 staining in lung from post-mortem sample of a patient who died from acute COVID-19 infection (SARS-CoV-2 antibody, Cell Signalling Technology; ×100 magnification); **c** IHC staining of endothelial SARS-CoV-2 expression in bowel mucosa of a patient in substudy 3 (SARS-CoV-2 antibody, Cell Signalling Technology; ×100 magnification, haematoxylin counterstain).

Specimens from 21 patients with a positive PCR test were analysed for SARS-CoV-2 and ACE-2 receptor expression in mesothelial fatty cells. All 21 patients had undergone emergency gastrointestinal surgery, and 8 had a perforation of the gastrointestinal tract at the time of surgery (*Table 2*). All samples were negative for SAR-CoV-2. One large bowel specimen was focally positive for ACE-2 receptor expression which was seen faintly in adipose cells only adjacent to the mesothelial cells (*Fig. 1a*). Luminal expression of the ACE-2 receptor was noted in large and small bowel samples, as expected (*Fig. S1C*).

**Table 2:** Clinical and surgical characteristics of patients in substudy 2, and summary of immunohistochemical analysis of SARS-CoV-2 and angiotensin-converting enzyme 2 receptor expression

	Substudy 2 (*n* = 21)		
**Age (years), median (range)**	57 (19–88)		
**Sex ratio (M : F)**	11:10		
**Positive PCR**	21		
**Time to surgery (days), median (range)**	1.5 (0–18)		
**Gastrointestinal perforation**	8		
**Surgical approach**			
Open	16		
Laparoscopic	5		
**Retrospective immunohistochemical analysis**	***n*** *	**SARS-Co-V2-positive**	**ACE-2-positive**
Colon	9 (4)	0 of 9	1 of 9 (weakly positive)
Small bowel/appendix	7 (2)	0 of 7	0 of 7
Hepatobiliary	3 (2)	0 of 3	0 of 3
Omentum	2	0 of 2	0 of 2

*Values in parentheses are number of patients with perforation. No virus or receptor expression was observed on peritoneal surfaces, other than weak focal expression in adjacent adipose cells from a single patient only.

### Substudy 3: post-mortem tissue samples

Samples were collected from 8 individuals with PCR-confirmed SAR-CoV-2. The serous membranes overlying abdominal and thoracic organs were examined for the presence of SARS-CoV-2 virus or ACE-2 receptor. None of the serous membranes examined by IHC showed evidence of virus protein or receptor expression (*Table 3*).

**Table 3:** Proportion of retrospective post-mortem samples in substudy 3 showing immunohistochemical detection of SARS-CoV-2 virus protein and angiotensin-converting enzyme 2 expression in post-mortem tissues from multiple organs in patients who died from COVID-19

	Substudy 3 (*n* = 8)	
**Age (years), median (range)**	74 (57–89)	
**Sex ratio (M : F)**	3 : 5	
**Retrospective immunohistochemical analysis**	**SARS-CoV-2-positive**	**ACE-2-positive**
Serous membrane		
Pleura	0 of 8	0 of 8
Pericardium	0 of 8	0 of 8
Peritoneum	0 of 4	0 of 4
Organs		
Lung	4 of 8	0 of 8
Heart	0 of 8	0 of 8
Liver	0 of 4	0 of 4
Pancreas	0 of 1	0 of 1
Gallbladder	0 of 1	0 of 1
Stomach	0 of 4	0 of 4
Small bowel	0 of 4	0 of 4
Large bowel	1 of 4 (endothelial only)	0 of 4
Uterus	0 of 1	0 of 1
Prostate	0 of 2	0 of 2

No virus or angiotensin-converting enzyme (ACE) 2 receptor expression was detected in serous membranes. Positive virus staining was observed in half of the lung samples examined and a large bowel blood vessel. Positive ACE-2 receptor staining was detected in the lumen of control bowel tissue, as expected.

The analysis was extended to the abdominal and thoracic organs of the 8 patients. Four of 8 lung samples showed positive IHC staining for SARS-CoV-2 nucleocapsid protein (*Fig. 1b*). Of these, 2 showed florid cytoplasmic staining in lung epithelium and macrophages, and 2 showed occasional staining of macrophages. Three of these 4 positive samples were from patients who died early in the disease course. Virus was not detected in any other organs tested, apart from a large bowel sample that showed positive staining for SARS-CoV-2 nucleocapsid protein in the endothelium of a small blood vessel just beneath the peritoneum; this staining was localized to a single vessel and is of uncertain significance (*Fig. 1c*).

## Discussion

Most reports regarding SARS-CoV-2 expression in the peritoneum have been from single or small case series, the largest^7^ documenting 34 of 65 patients with positive nasopharyngeal swabs undergoing caesarean section, and a more recent report^12^ of 13 patients undergoing emergency gastrointestinal surgery. Importantly, none of the patients in that series had perforation of the gastrointestinal tract. Of the 39 patients in the SAFE SURGERY study, 38 had confirmed nasopharyngeal swabs before surgery or autopsy; only 1 patient was included based on clinical suspicion only (recruited before PCR testing became standard of care).

No significant mesothelial expression of the ACE-2 receptor was observed, other than faint expression in adipose tissue; this was likely to be non-specific and of little significance. ACE-2 receptor expression was not observed in the lungs of post-mortem tissues, similar to the finding of other comprehensive studies reporting that receptor expression is often difficult to detect in the lungs^2^. Importantly, SARS-CoV-2 was not detected in peritoneal fluid nor on peritoneal surfaces of the wide range of intra-abdominal tissues assessed, including those from patients in whom there was a breach of the gastrointestinal tract. The latter result is consistent with a previous study^13^ that did not detect viral RNA in abdominal fluids.

Interestingly, SARS-CoV-2 nucleocapsid protein was detected in the endothelium of a blood vessel underlying the bowel mucosa of a patient who had died from acute COVID-19 infection. Viral RNAemia occurs during infection^14^ and, although no study has yet cultured live virus from blood, infrequent cases of COVID-19 placentitis (and the virus-positive blood vessel in the present study) suggest that extrapulmonary dissemination is possible. Importantly, COVID-19 placentitis is rare even though placental tissues express abundant ACE-2 protein^2^. The consistent lack of virus in the abdominal cavity and ACE-2 expression on peritoneal surfaces revealed in this and other studies suggests that the risk of viral transmission during surgery is very low. Although each substudy was relatively small, the unique combination of all three provides strong complementary evidence to justify these conclusions, and should also remain relevant for future variants or surges.

The study will allow informed decisions to be made regarding the use of personal protective equipment and filtration devices during these procedures. There are, of course, other potential routes whereby healthcare workers could be exposed to the virus, and it would appear sensible to continue to recommend careful patient assessment and that appropriate personal protection be employed according to local risk assessment and community levels of infection^15^.

## Supplementary Material

znac297_Supplementary_Data
