# Supplementary figures and images for: SARS-CoV-2 in the abdomen or pelvis: SAFE SURGERY study
Source: Br J Surg. 2022 Sep 28;110(3):306–9. doi: 10.1093/bjs/znac297 (PMC9620655; doi:10.1093/bjs/znac297)

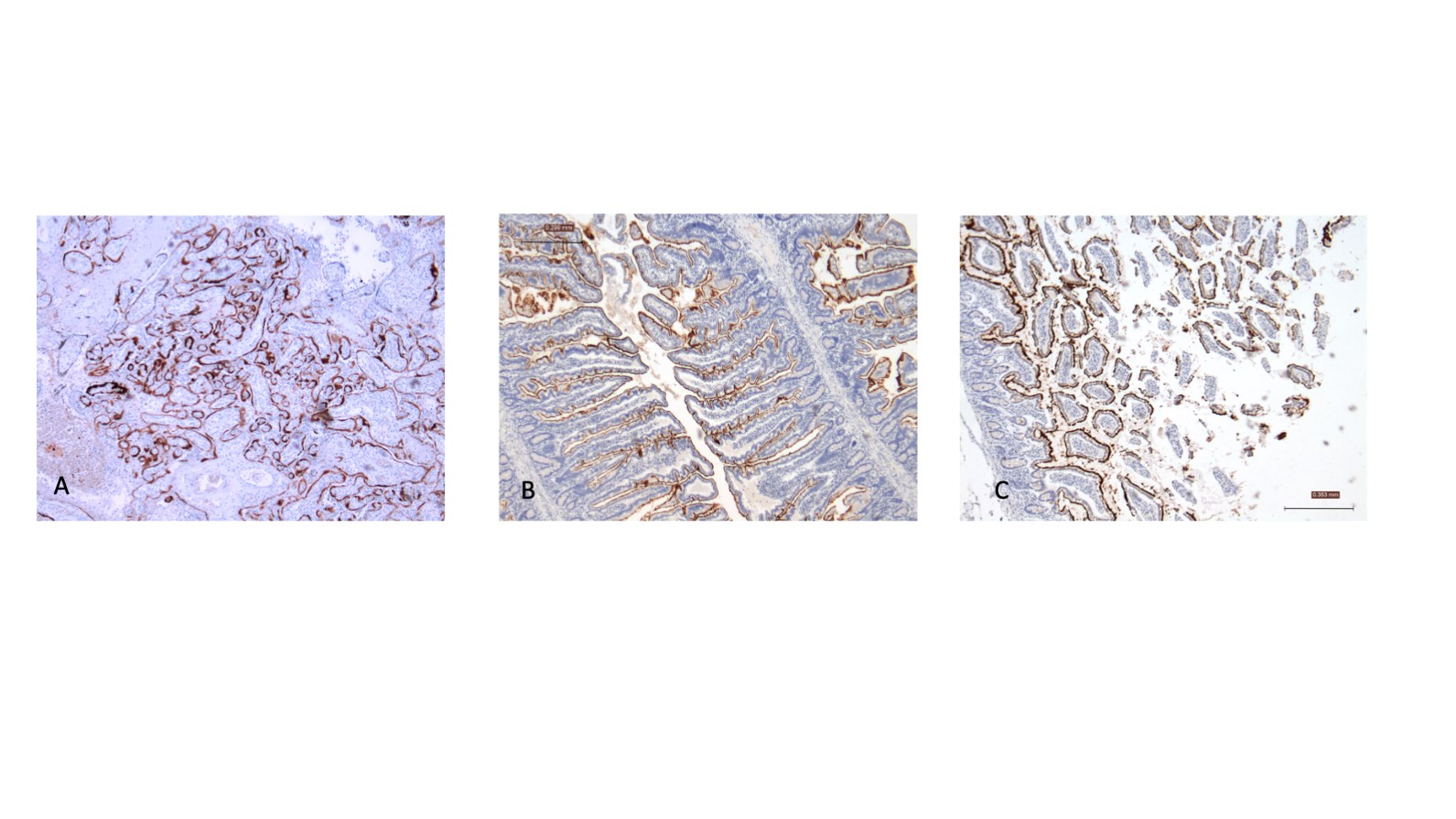

Supplement: znac297_Supplementary_Data [file znac297_supplementary_data.jpeg]
